# Systematic review of repetitive transcranial magnetic stimulation for post-stroke hemiplegic shoulder pain

**DOI:** 10.1007/s10072-024-07961-3

**Published:** 2025-01-02

**Authors:** Zhenchao Ma, Huijuan Pan, Ranran Bi, Zhenhua Li, Weichen Lu, Ping Wan

**Affiliations:** 1 School of Rehabilitation Medicine, Shanghai University of Traditional Chinese Medicine, Shanghai, 201203 China; 2 Department of Rehabilitation Medicine, Shanghai Ruijin Rehabilitation Hospital, Shanghai, 200023 China; 3 Department of Rehabilitation Medicine, Shanghai East Hospital, School of Medicine, Tongji University, Shanghai, 200120 China; 4 No.1200 Cailun Road, Pudong New Area, Shanghai, 10124 China

**Keywords:** Hemiplegic Shoulder Pain, Repetitive transcranial magnetic stimulation, Pain score, Upper Limb function, Systematic review and Meta-analysis

## Abstract

**Background:**

Repetitive transcranial magnetic stimulation (rTMS) has shown potential in alleviating hemiplegic shoulder pain (HSP) and improving upper limb function, yet its efficacy remains debated. This study aims to assess the effectiveness of rTMS for HSP through a systematic review and meta-analysis.

**Methods:**

Four databases were searched with the keywords “rTMS” and “HSP”. Adults aged 18 years and older with post-stroke HSP were included. The primary outcomes were pain scores and upper limb function scores, and the secondary outcomewas the incidence of adverse events. The risk of bias was assessed through the ROB tool in Review Manager 5.4.1, and statistical analysis was primarily conducted through this software.

**Results:**

A total of 52 articles were identified from PubMed, Embase, Cochrane Library, and CNKI. Following literature screening, 11 studies were included in the analysis. The quality of the included studies was moderate.The studies encompassed 584 patients with post-stroke HSP and their average age was 62. The analysis revealed that rTMSwas significantly more effective in relieving pain compared to the control group (SMD = -1.14, *p* < 0.01), and low-frequency rTMSwas superior to high-frequency rTMS. In terms of improving upper limb function, rTMSwas also significantly more efficacious compared to the control group (SMD = 2.20, *p* < 0.01), and low-frequency and high-intensity rTMSwere more beneficial.

**Conclusions:**

This study highlights the potential efficacy of rTMS. However, the heterogeneity among included studies, limited sample sizes, and lack of long-term follow-up data restrict the generalizability of the results.

**Supplementary Information:**

The online version contains supplementary material available at 10.1007/s10072-024-07961-3.

## Introduction

Stroke is one of the top three causes of increased mortality rates globally [1]. Hemiplegic shoulder pain (HSP) is a common complication among stroke survivors, affecting approximately 30-70% of this population [2]. HSP not only severely impacts patients’ quality of life but also complicates rehabilitation efforts and adds economic burden [3]. Patients with HSP often experience severe shoulder pain and restricted upper limb mobility, leading to a decrease in activities of daily living. The pathogenesis of HSP is multifactorial, involving both central nervous system-related and local lesions. Local lesions include shoulder subluxation, complex regional pain syndrome, biceps tendinitis, rotater cuff tears, adhesive capsulitis, and increased muscle tension. Central nervous system-related causes may share some common and complex mechanisms, involving abnormal neurotransmitter release, neuroinflammatory responses, changes in neuroplasticity, and mood disorders [4]. The complexity and variability of these mechanisms make the treatment of HSP particularly challenging.

Repetitive transcranial magnetic stimulation (rTMS), a non-invasive brain stimulation technique, has recently demonstrated significant potential in the field of neurological rehabilitation. rTMS has been shown to promote neuroplasticity and functional recovery [5–7], and alleviate stroke-related pain and mood disorders [8–10], offering substantial clinical benefits. Despite evidence suggesting that rTMS has some efficacy for HSP, there remains controversy regarding its effectiveness and mechanisms. Some studies indicate that rTMS can significantly alleviate HSP [11], while others have failed to observe notable effects [12]. Some research supports that rTMS improves HSP by enhancing neuroplasticity, promoting the reconstruction of neural networks, and reducing pain while improving motor function [13]. Other studies propose that rTMS alleviates pain by modulating neurotransmitter release (e.g., glutamate, GABA) or suppressing neuroinflammatory responses, though these mechanisms are not uniformly agreed upon [14].

This study, therefore, employed a systematic review and meta-analysis to comprehensively evaluate the effectiveness of rTMS in treating post-stroke HSP and aims to address inconsistencies in existing research. Furthermore, through the analysis of different frequencies, intensities, and treatment parameters of rTMS, the study seeks to identify the optimal regimen for alleviating HSP pain and improving upper limb function, providing evidence-based recommendations for clinical practice.

## Materials and methods

### Study registration and adherence to PRISMA guidelines

This study was performed in accordance with PRISMA guidelines [15] and pre-registered with PROSPERO (Registration Number: CRD42024525376).

### Literature search

A comprehensive search was carried out across PubMed, Embase, Cochrane Library, and China National Knowledge Infrastructure (CNKI) databases. The primary search terms included “rTMS” and “HSP,” with the search covering the period from database inception to June 2, 2024. No language or other restrictions were applied. The search history is provided in Table S1-4.

### Literature screening

Two independent reviewers conducted the literature screening, and a third expert consolidated the results. The inclusion criteria were as follows: (1) Patients had post-stroke HSP and were 18 years or older; (2) Intervention measures were rTMS, irrespective of frequency, duration, or location; (3) The control group received treatment strategies without rTMS, including but not limited to sham rTMS, acupuncture, intramuscular efficacy patch therapy, cold water immersion, rehabilitation training, extracorporeal shock wave therapy (ESWT), or laser therapy. For studies where non-sham rTMS was the control, the control group must have received the same background treatment as the rTMS group, ensuring comparable treatment conditions. (4) Primary outcomes included pain scores and upper limb function scores, while the secondary outcome was adverse events. (5) Only randomized controlled trials (RCTs) were included. Exclusion criteria were: (1) Full texts cannot be assessed; (2) Conference abstracts, communications, or other non-peer-reviewed sources.

### Risk of bias assessment

The risk of bias was assessed independently by two reviewers, with a third reviewer consolidating the results. The Review Manager 5.4.1 ROB tool was utilized for this assessment. The risk of bias was evaluated across seven domains: (1) random sequence generation; (2) allocation concealment; (3) blinding of participants; (4) blinding of outcome assessors; (5) incomplete outcome data; (6) selective reporting; (7) other sources of bias. Each domain was categorized as unclear, low, or high risk. The overall quality of studies was rated as low, moderate, or high based on all these domains.

### Data extraction

Data were extracted independently by two reviewers, and a third reviewer consolidated the data. A data extraction form was pre-designed. Key extracted information included: (1) study detasupils such as first author, methodology, year, and country; (2) population characteristics including sample size, gender, age, and disease severity; (3) outcome measures, with pain scores and upper limb function scores extracted as mean and standard deviation.

### Statistical analysis

Data analysis was primarily conducted through Review Manager 5.4.1. The heterogeneity of pooled estimates was quantified through the *I²* statistic; a random-effects model was employed if *I²* > 50% and *P* < 0.05; otherwise, a fixed-effects model was used. For continuous outcomes, the standard mean difference (SMD) was used to account for inherent differences in pain and upper limb function scores across studies. 95% confidence intervals (CI) were also calculated. Sensitivity analysis was performed if *I²* > 50% and *P* < 0.05. Additionally, publication bias was assessed through funnel plots and Egger’s test if more than ten studies were included. Since Egger’s test could not be performed in Review Manager, R was used for supplementary analysis as needed.

## Results

### Literature retrieval

**Fig. 1:**
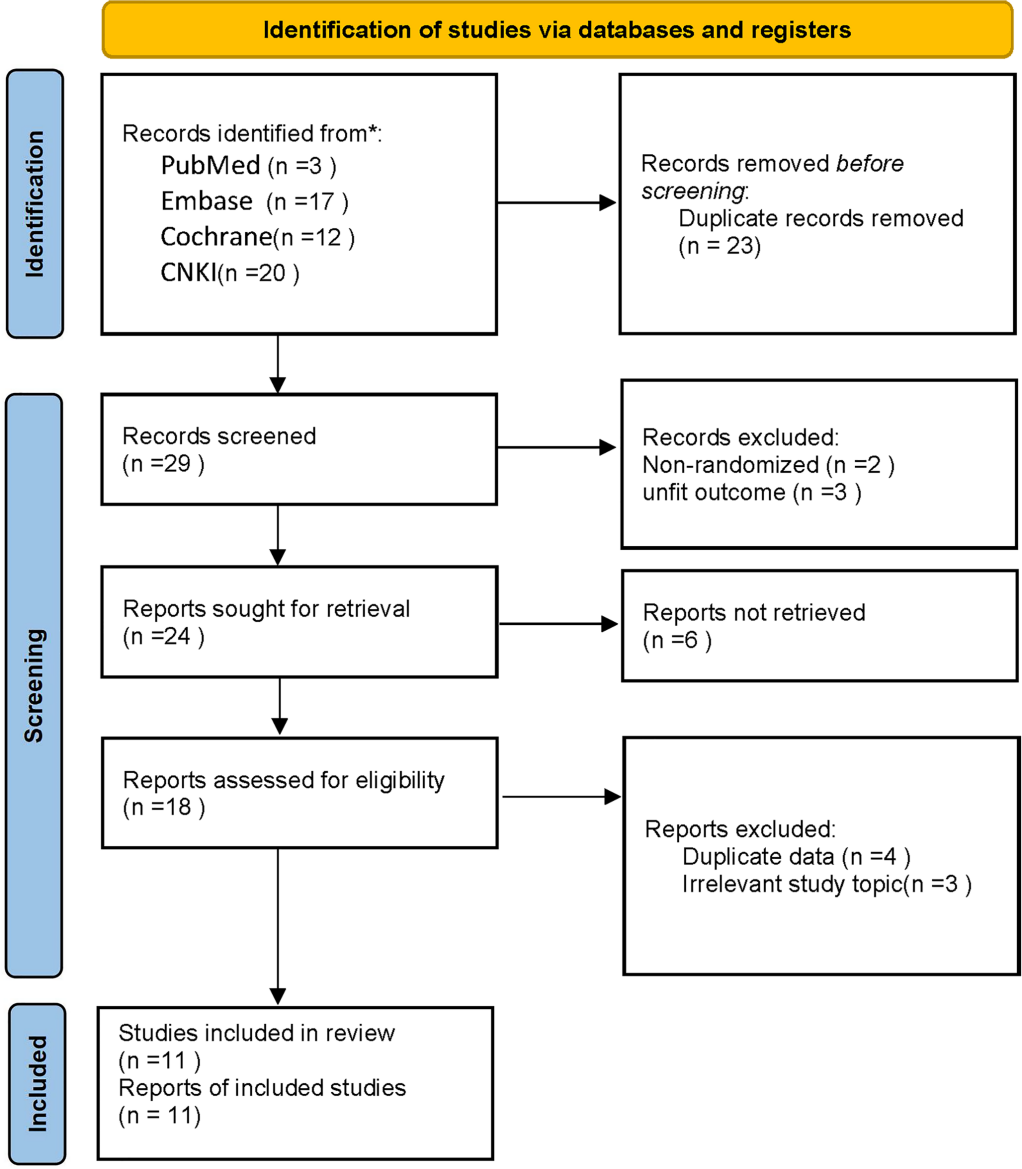
Flow chart of literature screening

A total of 52 articles were selected from PubMed, Embase, Cochrane Library, and CNKI. After duplication removal and initial screening, 18 articles remained. Upon full-text review, seven studies were excluded due to significant methodological flaws, inadequate data analysis, or irrelevance to the current research. Ultimately, 11 studies were included in the analysis (Fig. 1).

### Risk of bias and study characteristics

**Fig. 2:**
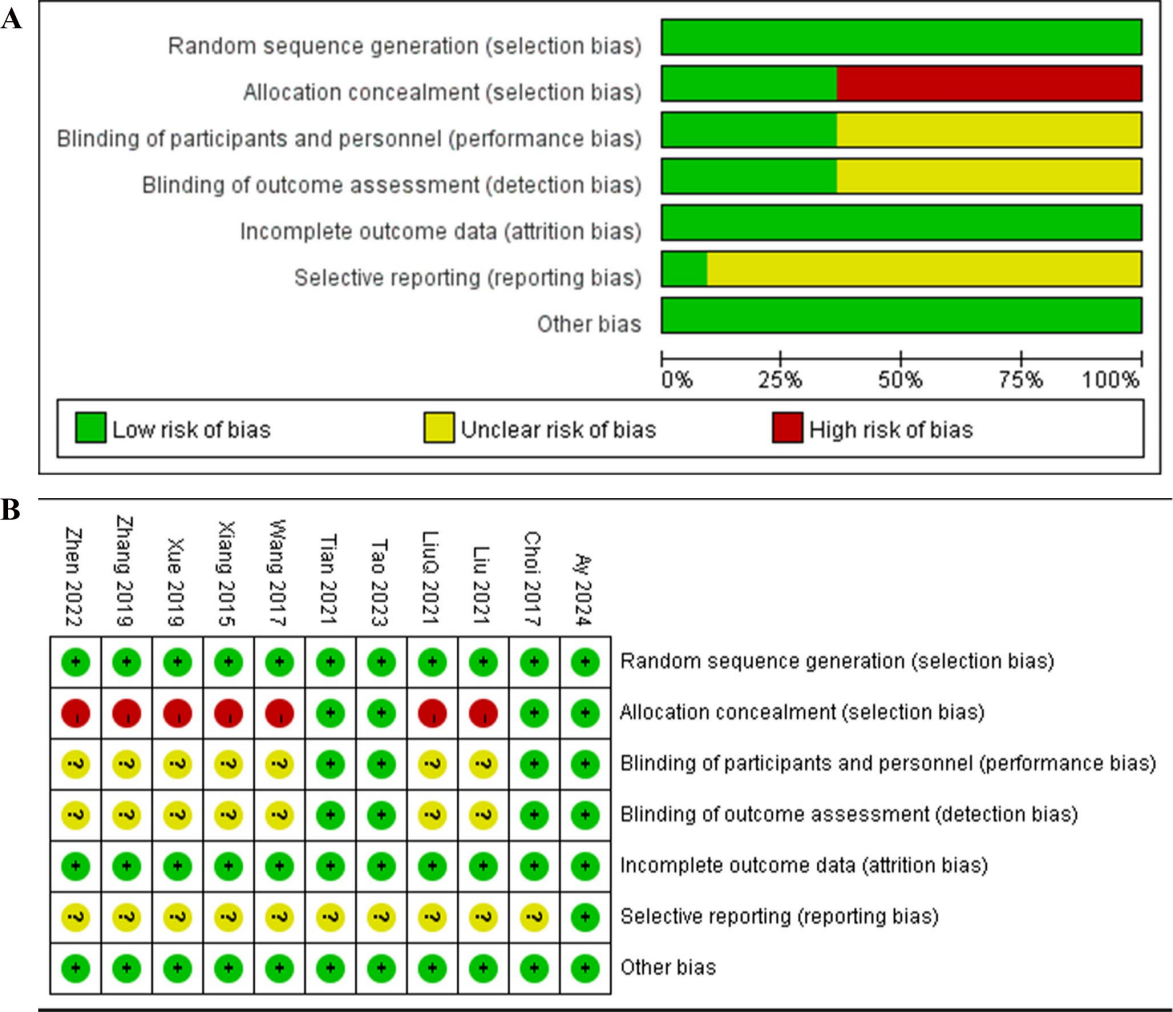
Risk of bias

Overall, the quality of the included studies was moderate. Major quality concerns encompassed: (1) inadequate description of allocation concealment; (2) lack of registration information in some studies, making it difficult to assess study quality and applicability; (3) small sample sizes leading to insufficient statistical power to support the research hypotheses (Fig. 2).

The included studies involved 584 patients with unilateral hemiplegia due to cerebral infarction, with an average age of 62, and a baseline numerical pain rating scale (NPRS) score ≥ 4. Of the included studies, five employed low-frequency rTMS, five used high-frequency rTMS, eight targeted the affected side, three targeted the unaffected side, six used low-intensity (≤ 80% RMT), and four used high-intensity (> 80% RMT) rTMS. Seven studies involved more than ten treatment sessions, while four studies had 10 or fewer sessions (Table 1). In particular, we analyzed the inclusion and exclusion criteria reported in eligible studies. As summarized in Tables 1 and 10 studies excluded focal causes of HSP, while the study by Xue 2019 did not mention it. The criteria for enrolling participants of each study are shown in Table S5.

**Table 1:** Demographic characteristics of included trials

Study	Group	Sample Size	Age (years, mean, SD)	Sex (M, F)	HSP Regional Etiology Excluded	Baseline Pain Score	Maximum Field Strength (T)	Stimulation Intensity	Frequency (Hz)	Stimulation Site	Duration	Course
Choi 2017	rTMS	12	60.30, 7.10	7, 5	Yes	NRS: 6.3, 1.3	1	90%RMT	10	Ipsilesional M1	20 min	qd, 2w, 10 times
	Sham	12	57.80, 8.90	6, 6		NRS: 5.8, 1.5						
Liu 2021	SGB	30	56.13,8.17	15,15	Yes	SF-MPQ:18.27,5.66	6	80%RMT	3	Ipsilesional M1	20 min	qd, 4w, 20 times
	rTMS&SGB	30	54.27,7.38	14,16		SF-MPQ:18.27,5.79						
LiuQ2021	CWBT	50	62.70,10.70	27,23	Yes	VAS:7.3,1.2	NR	NR	> 5	Contralateral M1	20 min	qd
	rTMS	50	60.20,10.80	29,21		VAS:7.1,1.3						
Tao 2023	Sham	20	57.85,3.17	8,12	Yes	NRS:7.25,1.55	2.5	80%RMT	10	Ipsilesional M1	20 min	qd, 4w, 20 times
	rTMS	20	58.85,2.90	11,9		NRS:6.85,1.50						
Tian 2021	Sham	29	65.90,9.50	14,15	Yes	VAS:6.18,1.59	NR	80%RMT	5	Ipsilesional DLPFC	20 min	qd, 2w, 12 times
	rTMS	28	65.97,10.51	15,13		VAS:6.00,1.39						
Wang 2017	AT	20	65.45,11.12	10,10	Yes	VAS:6.80,0.95	2	100%RMT	1	Contralateral M1	20 min	qd, 4w, 20 times
	rTMS&AT	20	62.13,12.54	11,9		VAS:6.85,0.99						
Xiang 2015	rTMS&Rb	28	52.20,	15,13	Yes	VAS:7.64,0.42	2.2	80%RMT	1	Ipsilesional M1	35 min	qd, 10 times
	rTMS	26	53.80,	16,10		VAS:8.07,1.76						
Xue 2019	rTMS&IEPT	35	NR	NR	No	NR	2	80%RMT	20	Ipsilesional M1	12.5 min	qd, 5 times
	IEPT	35	NR	NR		NR						
Zhang 2019	AT	30	64.82,5.87	14,16	Yes	VAS:6.32,0.24	2	100%RMT	1	Contralateral M1	20 min	qd, 4w, 20 times
	rTMS&AT	30	63.18,4.17	16,14		VAS:6.26,0.41						
Zhen 2022	Rb	30	64.03,3.12	20,10	Yes	VAS:5.49,1.10	2	80%RMT	1	Ipsilesional M1	35 min	qd, 4w, 20 times
	rTMS&Rb	30	64.39,3.08	18,12		VAS:5.32,1.03						
Ay 2024	rTMS	7	63	4,3	Yes	NRS: 6–8	1	90%RMT	5	Ipsilesional, M1	20 min	qd, 3w, 15 times
	Sham	11	66	7,4		NRS: 6–10						

Abbreviation: rTMS, repetitive transcranial magnetic stimulation; SGB, stellate ganglion block; CWBT, cold-water bathing therapy; AT, acupuncture therapy; IEPT, intramuscular efficacy patch therapy; Rb, rehabilitation; SD, Standard Deviation; M, male; F, female; NR, Not Reported; NRS, Numeric Rating Scale; RMT, Resting Motor Threshold; qd, quaque die; SF-MPQ, Short-Form McGill Pain Questionnaire; VAS, Visual Analogue Scale

### Statistical analysis results

**Fig. 3:**
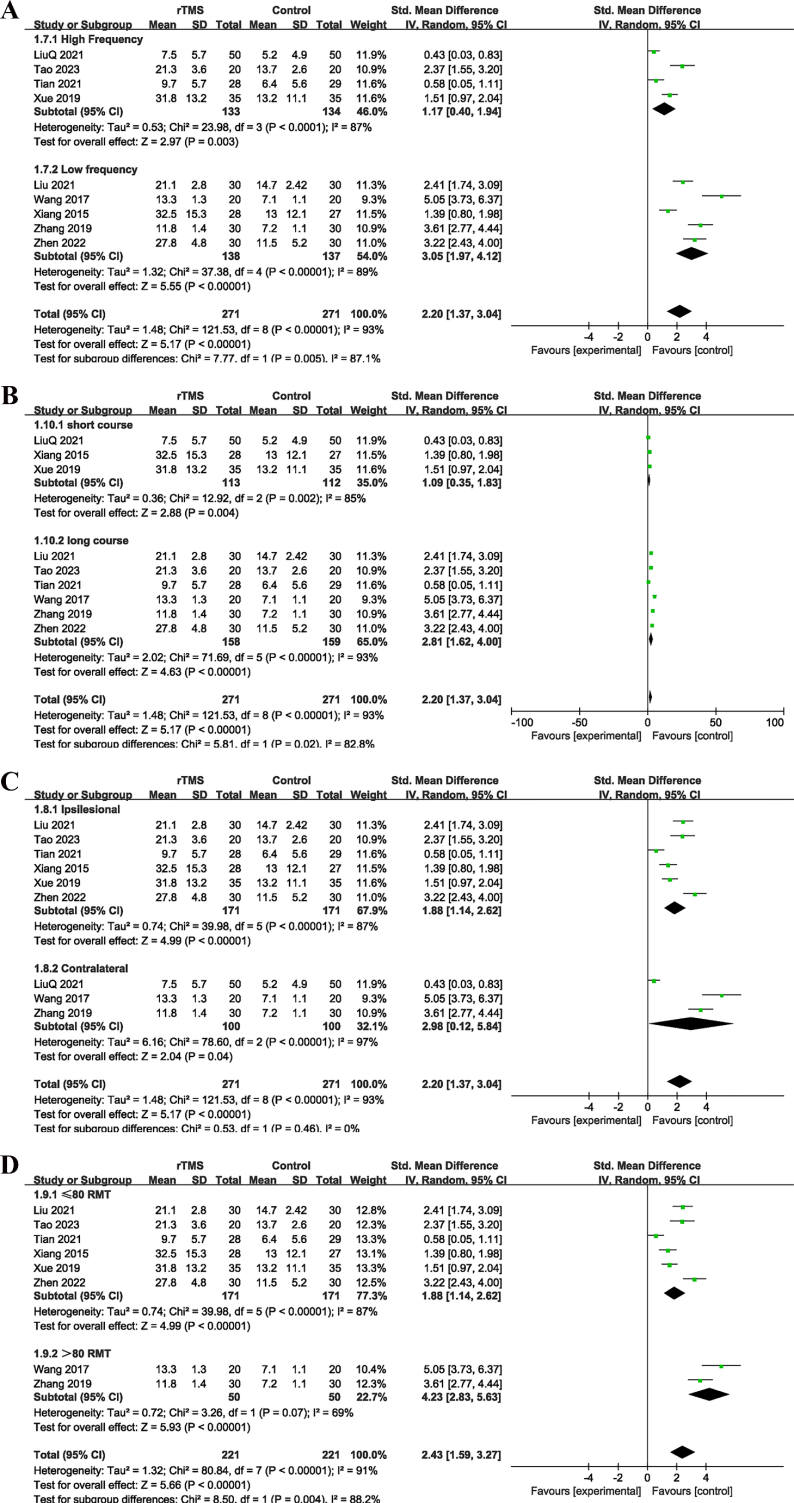
(**A**) Subgroup analysis of different treating frequencies in pain score; (**B**)Subgroup analysis of different treating courses in pain score; (**C**) Subgroup analysis of different treating sides in pain score; (**D**) Subgroup analysis of different treating intensity in pain score

In the analysis of pain scores, 10 studies involving 514 patients were included. Results indicated a significant difference between the rTMS group and the control group (*I²* = 87%, *P* < 0.01; random-effects model; SMD = -1.14 [-1.69, -0.58], *P* < 0.01; Fig. 3). Subgroup analysis revealed that low-frequency rTMS (-1.67 [-2.69, -0.65]) was significantly more effective than high-frequency rTMS (-0.60 [-0.91, -0.28]) (*P* < 0.01). No significant differences were observed between unaffected and affected sides, different stimulation intensities, or different sessions of treatments. Sensitivity analysis confirmed the stability of the results. Egger’s test yielded a *P*-value of 0.12, suggesting no significant publication bias.

**Fig. 4:**
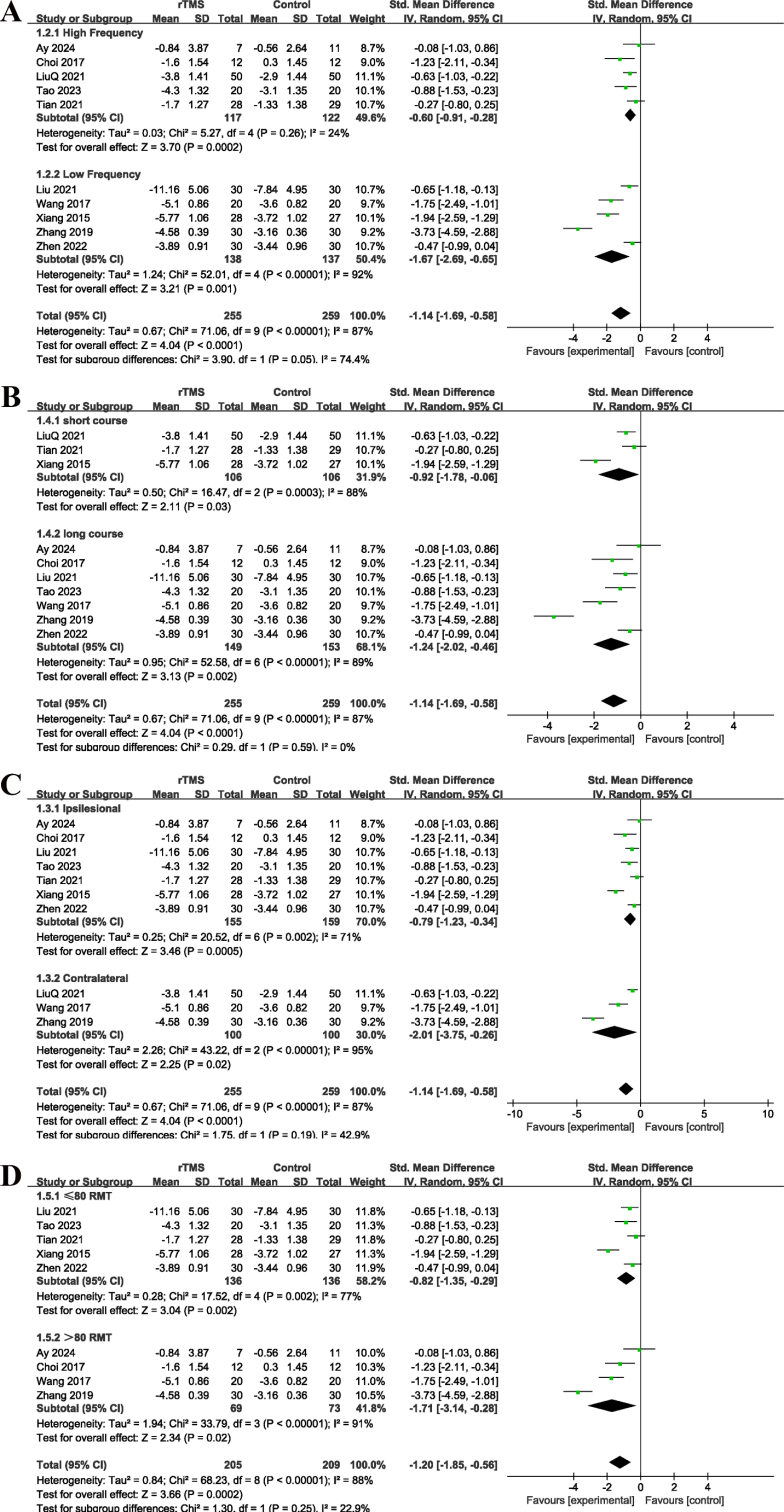
(**A**) Subgroup analysis of different treating frequencies in Upper Limb Function; (**B**) Subgroup analysis of different treating courses in Upper Limb Function; (**C**) Subgroup analysis of different treating sides in Upper Limb Function; (**D**) Subgroup analysis of different treating intensity in Upper Limb Function

In the analysis of upper limb function, nine studies involving 542 patients were included. Results demonstrated a significant difference between the rTMS group and the control group (*I²* = 93%, *P* < 0.01; random-effects model; SMD = 2.20 [1.37, 3.04], *P* < 0.01; Fig. 4). Subgroup analysis indicated that low-frequency rTMS (3.05 [1.97, 4.12]) was significantly more effective than high-frequency rTMS (SMD = 1.17 [0.40, 1.94]) (*P* < 0.01). The high-intensity group (> 80% RMT, SMD = 4.23 [2.83, 5.63]) also showed significantly better outcomes compared to the low-intensity group (≤ 80% RMT, SMD = 1.88 [1.14, 2.62]) (*P* < 0.01). No significant differences were found among different stimulation sites or treatment sessions. Sensitivity analysis was performed to validate the stability of the results. Due to fewer than 10 studies, Egger’s test was not performed.

## Discussion

This study demonstrates that rTMS significantly relieves pain and improves upper limb motor function in patients with post-stroke HSP. Additionally, treatment frequency and intensity may influence the efficacy of rTMS.

The primary etiological factors contributing to HSP include abnormal neurotransmitter release, neuroinflammatory responses, changes in neuroplasticity, and mood disorders. Research indicates that neurotransmitters such as glutamate and GABA are abnormally released in the central nervous system (CNS) of stroke patients, a phenomenon associated with the inhibition of pain perception [16, 17]. Previous studies have shown that rTMS can modulate the function of GABAergic neurons through cortical inhibition, thereby ameliorating pain symptoms [18–20]. Therefore, the relief of HSP pain by rTMS may be attributed to its ability to suppress the abnormal release of neurotransmitters. Local and systemic inflammatory responses, characterized by elevated levels of tumor necrosis factor-alpha (TNF-α) and interleukin-6 (IL-6), have been well-established in post-stroke patients. Microglia, astrocytes, and neurons, affected by these inflammatory responses, may heighten pain perception [16, 21]. Studies have proved that rTMS reduces neuroinflammation by inhibiting microglial polarization and decreasing inflammatory cytokines (e.g., TNF-α, IL-1β, and IL-6), thus alleviating pain symptoms in HSP patients [22]. Neuroplasticity is closely linked to early neural repair following stroke and subsequent symptoms and functional outcomes [23–25]. rTMS has been demonstrated to activate connections between different parts of the brain’s signal network, increase serum levels of brain-derived neurotrophic factor (BDNF), and modulate long-term potentiation (LTP) and long-term depression (LTD) mechanisms [26–30]. Therefore, the alleviation of HSP pain by rTMS may result from its effects on enhancing neuroplasticity. HSP is frequently accompanied by depression and anxiety, which exacerbate pain and hinder the rehabilitation process. Studies have indicated that rTMS not only alleviates central post-stroke pain (CPSP) but also improves accompanying depressive and anxious symptoms [31]. Thus, rTMS may enhance pain perception in patients by improving emotions.

The primary causes of upper limb dysfunction in HSP patients include shoulder joint subluxation, adhesive capsulitis (frozen shoulder), and neuropathic pain. Research has shown a correlation between shoulder joint subluxation and upper limb dysfunction in HSP [32]. Reduced movement in hemiplegic patients causes stiffness in the muscles, tendons, and ligaments surrounding the shoulder joint, which ultimately restricts the movement. Weakness of shoulder muscles, particularly levator scapulae, trapezius, and deltoid, compromises shoulder joint stability, thereby leading to subluxation, pain, and limited range of motion [33]. Previous studies suggest that rTMS may indirectly reduce the incidence and severity of shoulder joint subluxation by improving neural function, muscle strength, and coordination [34, 35]. Thus, the improvement in upper limb function in HSP patients due to rTMS may be ascribed to increased shoulder muscle strength and stability, which reduces shoulder joint subluxation. The incidence of adhesive capsulitis and its impact on upper limb function in stroke patients have been well-documented [36, 37]. Studies have found that high-frequencyr TMS can effectively relieve chronic pain [20]. For patients with adhesive capsulitis, rTMS may improve upper limb functional activities by alleviating pain and inflammation [38]. Research evaluating the role of neuropathic pain in post-stroke shoulder pain and its impact on shoulder joint function has highlighted the restrictive effects of chronic pain on joint mobility [39]. Neuropathic pain not only directly causes pain but also indirectly leads to restricted joint movement by affecting patients’ willingness to move and their movement patterns [40, 41]. rTMS has been reported to modulate cortical excitability and neural network activity to alleviate chronic pain, including neuropathic pain [42]. This mechanism is particularly relevant for HSP treatment, as HSP is commonly associated with chronic pain. By altering the processing of pain signals in the brain, rTMS helps to reduce pain and improve the comfort and function of upper limb joint movements [43, 44].

Among the studies included in our review, most utilized devices provided by RAPID-II. The primary motor cortex (M1) is the most commonly targeted region used for addressing both pain and motor impairments. Our findings suggest that lower frequencies and higher treatment intensities may be associated with better therapeutic outcomes. Different frequencies and intensities of rTMS are believed to have distinct effects. Low-intensity rTMS and low-frequency rTMS are commonly employed to inhibit hyperexcited neuronal activity. This inhibitory effect can help alleviate pain by reducing the transmission of pain signals [10, 45]. Concurrently, low-intensity rTMS can achieve therapeutic effects by modulating neural network activity without excessively stimulating neurons, potentially making it more suitable for sensitive or easily provoked patients [46]. Conversely, high-intensity rTMS and high-frequency rTMS are typically used to enhance neuronal activity. This excitatory effect can promote neuroplasticity and functional recovery, such as improving upper limb function by activating the motor cortex [47]. The stronger neural stimulation provided by high-intensity rTMS might lead to more pronounced therapeutic effects but also comes with a higher risk of side effects [48]. A possible explanation for our findings is that rTMS may have multifaceted effects, rather than a singular inhibitory or excitatory effect. Low-frequency and high-intensity stimulation may improve function through different mechanisms. Various frequencies or intensities of rTMS may exert beneficial regulatory effects on neural networks at different levels. Additionally, in the included studies, Chinese researchers often employed low-intensity rTMS, while researchers outside of China more frequently used high-intensity rTMS. However, neither group provided specific justifications for the choice of different intensities. Given the potential impact of intensity on outcomes, further attention to this aspect is warranted in future research.

This systematic review pooled the data and findings from multiple studies. By analyzing rTMS parameters and therapeutic effects across different studies, this study elucidates the advantages and limitations of various parameter combinations, potentially aiding clinicians in making more informed decisions in practice. Nonetheless, there are several limitations in this study. First, the included studies exhibit heterogeneity in research design, treatment parameters, and assessment methods. Therefore, it is necessary to carefully consider this heterogeneity when interpreting and applying these findings. Future research may need to explore more refined assessment and monitoring methods for individualized treatments. Second, although this review incorporates data from multiple studies, the overall sample size remains insufficient to fully represent the HSP population. The limited sample size may affect the statistical significance and generalizability of the results. Future research should increase the sample size to enhance the reliability and representativeness of the findings. Lastly, most studies primarily focus on short-term treatment effects, with insufficient follow-up data on the long-term effects and safety of rTMS. Long-term side effects and tolerance issues need to be further investigated to ensure the safe application of rTMS in HSP treatment. In particular, the long-term impact of high-intensity rTMS needs to be further validated. Finally, although our study has demonstrated the benefits of low-frequency and high-intensity rTMS, further research is needed to verify our findings due to a limited number of available studies.

## Conclusion

This study indicates that rTMS is effective in relieving pain and improving upper limb motor function in patients with HSP. Additionally, lower frequencies and higher intensities of rTMS may provide greater therapeutic benefits. However, due to limitations such as the small sample size and significant differences in treatment protocols among the included studies, the findings should be interpreted with caution. Future clinical research on individualized treatment protocols with robust designs, larger sample sizes, and long-term follow-up is necessary.

## Electronic Supplementary Material

Below is the link to the electronic supplementary material.

Supplementary Material 1: Table **S1** Literature Search History of PubMed, Table **S2** Literature Search History of Embase, Table **S3** Literature Search History of Cochrane Library, Table **S4** China National Knowledge Infrastructure, Table **S5** The eligibility criteria of subjects in included trials.

## Data Availability

All data generated or analysed during this study are included in this published article and its supplementary information files.

## References

[1] Collaborators GDH (2017) Global, regional, and national disability-adjusted life-years (DALYs) for 333 diseases and injuries and healthy life expectancy (HALE) for 195 countries and territories, 1990–2016: a systematic analysis for the global burden of Disease Study 2016. Lancet 390(10100):1260–1344. 10.1016/s0140-6736(17)32130-x 28919118 10.1016/S0140-6736(17)32130-X PMC5605707

[2] Lindgren I, Jönsson AC, Norrving B, Lindgren A (2007) Shoulder pain after stroke: a prospective population-based study. Stroke 38(2):343–348. 10.1161/01.STR.0000254598.16739.4e 17185637 10.1161/01.STR.0000254598.16739.4e

[3] Ratnasabapathy Y, Broad J, Baskett J, Pledger M, Marshall J, Bonita R (2003) Shoulder pain in people with a stroke: a population-based study. Clin Rehabil 17(3):304–311. 10.1191/0269215503cr612oa 12735538 10.1191/0269215503cr612oa

[4] Coskun Benlidayi I, Basaran S (2014) Hemiplegic shoulder pain: a common clinical consequence of stroke. Pract Neurol 14(2):88–91. 10.1136/practneurol-2013-000606 23940374 10.1136/practneurol-2013-000606

[5] Kobayashi M, Pascual-Leone A (2003) Transcranial magnetic stimulation in neurology. Lancet Neurol 2(3):145–156. 10.1016/s1474-4422(03)00321-1 12849236 10.1016/s1474-4422(03)00321-1

[6] Tang Z, Han K, Wang R, Zhang Y, Zhang H (2022) Excitatory repetitive transcranial magnetic stimulation over the Ipsilesional Hemisphere for Upper Limb motor function after stroke: a systematic review and Meta-analysis. Front Neurol 13:918597. 10.3389/fneur.2022.918597 35795793 10.3389/fneur.2022.918597 PMC9251503

[7] Bai Z, Zhang J, Fong KNK (2022) Effects of transcranial magnetic stimulation in modulating cortical excitability in patients with stroke: a systematic review and meta-analysis. J Neuroeng Rehabil 19(1):24. 10.1186/s12984-022-00999-4 35193624 10.1186/s12984-022-00999-4 PMC8862292

[8] Gu SY, Chang MC (2017) The effects of 10-Hz repetitive transcranial magnetic stimulation on Depression in Chronic Stroke patients. Brain Stimul 10(2):270–274. 10.1016/j.brs.2016.10.010 27839722 10.1016/j.brs.2016.10.010

[9] Ricardo EJ, Robert GR, Amane T, Kenji N, Laura A, David M et al (2004) Repetitive transcranial magnetic stimulation as treatment of poststroke depression: a preliminary study. Biol Psychiatry 55(4):398–405. 10.1016/j.biopsych.2003.08.017 14960293 10.1016/j.biopsych.2003.08.017

[10] Kim YH, You SH, Ko MH, Park JW, Lee KH, Jang SH et al (2006) Repetitive transcranial magnetic stimulation-induced corticomotor excitability and associated motor skill acquisition in chronic stroke. Stroke 37(6):1471–1476. 10.1161/01.Str.0000221233.55497.51 16675743 10.1161/01.STR.0000221233.55497.51

[11] Choi GS, Kwak SG, Lee HD, Chang MC (2018) Effect of high-frequency repetitive transcranial magnetic stimulation on chronic central pain after mild traumatic brain injury: a pilot study. J Rehabil Med 50(3):246–252. 10.2340/16501977-2321 29392332 10.2340/16501977-2321

[12] Chen S, Zhou Z, Ren M, Chen X, Shi X, Zhang S et al (2023) Case report: high-frequency repetitive transcranial magnetic stimulation for treatment of hereditary spastic paraplegia type 11. Front Neurol 14:1162149. 10.3389/fneur.2023.1162149 37273711 10.3389/fneur.2023.1162149 PMC10232891

[13] Kimura I, Senoo A, Abo M (2024) Changes in structural neural networks in the recovery process of Motor Paralysis after Stroke. Brain Sci 14(3). 10.3390/brainsci14030197 10.3390/brainsci14030197 PMC10968532 38539586

[14] Stagg CJ, Nitsche MA (2011) Physiological basis of transcranial direct current stimulation. Neuroscientist 17(1):37–53. 10.1177/1073858410386614 21343407 10.1177/1073858410386614

[15] Page MJ, McKenzie JE, Bossuyt PM, Boutron I, Hoffmann TC, Mulrow CD et al (2021) The PRISMA 2020 statement: an updated guideline for reporting systematic reviews. BMJ 372:n71. 10.1136/bmj.n71 33782057 10.1136/bmj.n71 PMC8005924

[16] Hiu T, Farzampour Z, Paz JT, Wang EH, Badgely C, Olson A et al (2016) Enhanced phasic GABA inhibition during the repair phase of stroke: a novel therapeutic target. Brain 139(Pt 2):468–480. 10.1093/brain/awv360 26685158 10.1093/brain/awv360 PMC4805083

[17] Vuagnat H, Chantraine A (2003) Shoulder pain in hemiplegia revisited: contribution of functional electrical stimulation and other therapies. J Rehabil Med 35(2):49–54 quiz 56. 10.1080/16501970306111 12691333 10.1080/16501970306111

[18] Lanza G, Aricò D, Lanuzza B, Cosentino FII, Tripodi M, Giardina F et al (2020) Facilitatory/inhibitory intracortical imbalance in REM sleep behavior disorder: early electrophysiological marker of neurodegeneration? Sleep 43(3). 10.1093/sleep/zsz242 10.1093/sleep/zsz242 31599326

[19] Hosomi K, Kishima H, Oshino S, Hirata M, Tani N, Maruo T et al (2013) Cortical excitability changes after high-frequency repetitive transcranial magnetic stimulation for central poststroke pain. Pain 154(8):1352–1357. 10.1016/j.pain.2013.04.017 23707310 10.1016/j.pain.2013.04.017

[20] Lefaucheur JP, Drouot X, Ménard-Lefaucheur I, Keravel Y, Nguyen JP (2006) Motor cortex rTMS restores defective intracortical inhibition in chronic neuropathic pain. Neurology 67(9):1568–1574. 10.1212/01.wnl.0000242731.10074.3c 17101886 10.1212/01.wnl.0000242731.10074.3c

[21] Zhang X, Wang J, Zhou Q, Xu Y, Pu S, Wu J et al (2011) Brain-derived neurotrophic factor-activated astrocytes produce mechanical allodynia in neuropathic pain. Neuroscience 199:452–460. 10.1016/j.neuroscience.2011.10.017 22044922 10.1016/j.neuroscience.2011.10.017

[22] Bai YW, Yang QH, Chen PJ, Wang XQ (2023) Repetitive transcranial magnetic stimulation regulates neuroinflammation in neuropathic pain. Front Immunol 14:1172293. 10.3389/fimmu.2023.1172293 37180127 10.3389/fimmu.2023.1172293 PMC10167032

[23] Miller KJ, Hermes D, Honey CJ, Hebb AO, Ramsey NF, Knight RT et al (2012) Human motor cortical activity is selectively phase-entrained on underlying rhythms. PLoS Comput Biol 8(9):e1002655. 10.1371/journal.pcbi.1002655 22969416 10.1371/journal.pcbi.1002655 PMC3435268

[24] Schaechter JD (2004) Motor rehabilitation and brain plasticity after hemiparetic stroke. Prog Neurobiol 73(1):61–72. 10.1016/j.pneurobio.2004.04.001 15193779 10.1016/j.pneurobio.2004.04.001

[25] Nudo RJ (2006) Mechanisms for recovery of motor function following cortical damage. Curr Opin Neurobiol 16(6):638–644. 10.1016/j.conb.2006.10.004 17084614 10.1016/j.conb.2006.10.004

[26] Hallett M, Di Iorio R, Rossini PM, Park JE, Chen R, Celnik P et al (2017) Contribution of transcranial magnetic stimulation to assessment of brain connectivity and networks. Clin Neurophysiol 128(11):2125–2139. 10.1016/j.clinph.2017.08.007 28938143 10.1016/j.clinph.2017.08.007 PMC5679437

[27] Siotto M, Aprile I, Simonelli I, Pazzaglia C, Ventriglia M, Santoro M et al (2017) An exploratory study of BDNF and oxidative stress marker alterations in subacute and chronic stroke patients affected by neuropathic pain. J Neural Transm (Vienna) 124(12):1557–1566. 10.1007/s00702-017-1805-9 29086097 10.1007/s00702-017-1805-9

[28] Zhao CG, Sun W, Ju F, Jiang S, Wang H, Sun XL et al (2021) Analgesic effects of Navigated Repetitive Transcranial Magnetic Stimulation in patients with Acute Central Poststroke Pain. Pain Ther 10(2):1085–1100. 10.1007/s40122-021-00261-0 33866522 10.1007/s40122-021-00261-0 PMC8586137

[29] Huang Y, Lin R, Li H, Xu Y, Tian F, Ma L et al (2023) Protocol for a single-blind randomized clinical trial to test the efficacy of bilateral transcranial magnetic stimulation on upper extremity motor function in patients recovering from stroke. Trials 24(1):601. 10.1186/s13063-023-07584-7 37735708 10.1186/s13063-023-07584-7 PMC10515042

[30] Dall’Agnol L, Medeiros LF, Torres IL, Deitos A, Brietzke A, Laste G et al (2014) Repetitive transcranial magnetic stimulation increases the corticospinal inhibition and the brain-derived neurotrophic factor in chronic myofascial pain syndrome: an explanatory double-blinded, randomized, sham-controlled trial. J Pain 15(8):845–855. 10.1016/j.jpain.2014.05.001 24865417 10.1016/j.jpain.2014.05.001

[31] Galhardoni R, Aparecida da Silva V, García-Larrea L, Dale C, Baptista AF, Barbosa LM et al (2019) Insular and anterior cingulate cortex deep stimulation for central neuropathic pain: disassembling the percept of pain. Neurology 92(18):e2165–e2175. 10.1212/wnl.0000000000007396 30952795 10.1212/WNL.0000000000007396

[32] Zorowitz RD, Hughes MB, Idank D, Ikai T, Johnston MV (1996) Shoulder pain and subluxation after stroke: correlation or coincidence? Am J Occup Ther 50(3):194–201. 10.5014/ajot.50.3.194 8822242 10.5014/ajot.50.3.194

[33] Fitterer JW, Picelli A, Winston P (2021) A Novel Approach to New-Onset Hemiplegic Shoulder Pain with decreased range of Motion using targeted diagnostic nerve blocks: the ViVe Algorithm. Front Neurol 12:668370. 10.3389/fneur.2021.668370 34122312 10.3389/fneur.2021.668370 PMC8194087

[34] Khedr EM, Ahmed MA, Fathy N, Rothwell JC (2005) Therapeutic trial of repetitive transcranial magnetic stimulation after acute ischemic stroke. Neurology 65(3):466–468. 10.1212/01.wnl.0000173067.84247.36 16087918 10.1212/01.wnl.0000173067.84247.36

[35] Hummel FC, Cohen LG (2006) Non-invasive brain stimulation: a new strategy to improve neurorehabilitation after stroke? Lancet Neurol 5(8):708–712. 10.1016/s1474-4422(06)70525-7 16857577 10.1016/S1474-4422(06)70525-7

[36] Blennerhassett JM, Gyngell K, Crean R (2010) Reduced active control and passive range at the shoulder increase risk of shoulder pain during inpatient rehabilitation post-stroke: an observational study. J Physiother 56(3):195–199. 10.1016/s1836-9553(10)70025-4 20795926 10.1016/s1836-9553(10)70025-4

[37] Appelros P (2006) Prevalence and predictors of pain and fatigue after stroke: a population-based study. Int J Rehabil Res 29(4):329–333. 10.1097/MRR.0b013e328010c7b8 17106351 10.1097/MRR.0b013e328010c7b8

[38] Zhong J, Lan W, Feng Y, Yu L, Xiao R, Shen Y et al (2022) Efficacy of repetitive transcranial magnetic stimulation on chronic migraine: a meta-analysis. Front Neurol 13:1050090. 10.3389/fneur.2022.1050090 36504667 10.3389/fneur.2022.1050090 PMC9730425

[39] Turner-Stokes L, Jackson D (2002) Shoulder pain after stroke: a review of the evidence base to inform the development of an integrated care pathway. Clin Rehabil 16(3):276–298. 10.1191/0269215502cr491oa 12017515 10.1191/0269215502cr491oa

[40] Lepesis V, Paton J, Rickard A, Latour JM, Marsden J (2023) Effects of foot and ankle mobilisations combined with home stretches in people with diabetic peripheral neuropathy: a proof-of-concept RCT. J Foot Ankle Res 16(1):88. 10.1186/s13047-023-00690-4 38057930 10.1186/s13047-023-00690-4 PMC10699018

[41] Güngör Demir U, Demir AN, Toraman NF (2021) Neuropathic pain in knee osteoarthritis. Adv Rheumatol 61(1):67. 10.1186/s42358-021-00225-0 34743761 10.1186/s42358-021-00225-0

[42] Attia M, McCarthy D, Abdelghani M (2021) Repetitive Transcranial Magnetic Stimulation for treating Chronic Neuropathic Pain: a systematic review. Curr Pain Headache Rep 25(7):48. 10.1007/s11916-021-00960-5 33978846 10.1007/s11916-021-00960-5

[43] Dongyang L, Fernandes AM, da Cunha PHM, Tibes R, Sato J, Listik C et al (2021) Posterior-superior insular deep transcranial magnetic stimulation alleviates peripheral neuropathic pain - A pilot double-blind, randomized cross-over study. Neurophysiol Clin 51(4):291–302. 10.1016/j.neucli.2021.06.003 34175192 10.1016/j.neucli.2021.06.003

[44] Vabalaite B, Petruseviciene L, Savickas R, Kubilius R, Ignatavicius P, Lendraitiene E (2021) Effects of high-frequency (HF) Repetitive Transcranial Magnetic Stimulation (rTMS) on Upper Extremity Motor function in Stroke patients: a systematic review. Med (Kaunas) 57(11). 10.3390/medicina57111215 10.3390/medicina57111215 PMC8617907 34833433

[45] Lefaucheur JP, André-Obadia N, Antal A, Ayache SS, Baeken C, Benninger DH et al (2014) Evidence-based guidelines on the therapeutic use of repetitive transcranial magnetic stimulation (rTMS). Clin Neurophysiol 125(11):2150–2206. 10.1016/j.clinph.2014.05.021 25034472 10.1016/j.clinph.2014.05.021

[46] Chen R, Classen J, Gerloff C, Celnik P, Wassermann EM, Hallett M et al (1997) Depression of motor cortex excitability by low-frequency transcranial magnetic stimulation. Neurology 48(5):1398–1403. 10.1212/wnl.48.5.1398 9153480 10.1212/wnl.48.5.1398

[47] Kondo T, Yamada N, Momosaki R, Shimizu M, Abo M (2017) Comparison of the effect of Low-Frequency Repetitive Transcranial Magnetic Stimulation with that of Theta Burst Stimulation on Upper Limb Motor function in Poststroke patients. Biomed Res Int 2017:4269435. 10.1155/2017/4269435 29230407 10.1155/2017/4269435 PMC5694591

[48] O’Reardon JP, Solvason HB, Janicak PG, Sampson S, Isenberg KE, Nahas Z et al (2007) Efficacy and safety of transcranial magnetic stimulation in the acute treatment of major depression: a multisite randomized controlled trial. Biol Psychiatry 62(11):1208–1216. 10.1016/j.biopsych.2007.01.018 17573044 10.1016/j.biopsych.2007.01.018

