# Supplementary material for: Systematic review of repetitive transcranial magnetic stimulation for post-stroke hemiplegic shoulder pain
Source: Neurol Sci. 2025 Jan 2;46(5):2007–17. doi: 10.1007/s10072-024-07961-3 (PMC12003621; doi:10.1007/s10072-024-07961-3)
Supplement: Supplementary file 1 — Supplementary Material 1: Table S1 Literature Search History of PubMed, Table S2 Literature Search History of Embase, Table S3 Literature Search History of Cochrane Library, Table S4 China National Knowledge Infrastructure, Table S5 The eligibility criteria of subjects in included trials. [file 10072_2024_7961_MOESM1_ESM.docx]

**Supplementary Material**

## Table S1 Literature Search History of PubMed

| Pubmed | | |
| --- | --- | --- |
| # | Query | Results |
| 1 | "pain, shoulder"[Title/Abstract] OR "painful shoulder syndrome"[Title/Abstract] OR "Pains, Shoulder"[Title/Abstract] OR "shoulder pain"[Title/Abstract] OR "Shoulder Pains"[Title/Abstract] | 9608 |
| 2 | Shoulder Pain[MeSH Terms] | 6105 |
| 3 | "Acute Cerebrovascular Accident"[Title/Abstract] OR "Acute Cerebrovascular Accidents"[Title/Abstract] OR "acute cerebrovascular lesion"[Title/Abstract] OR "acute focal cerebral vasculopathy"[Title/Abstract] OR "acute stroke"[Title/Abstract] OR "Acute Strokes"[Title/Abstract] OR "Acute Stroke"[Title/Abstract] OR "apoplectic stroke"[Title/Abstract] OR "apoplexia"[Title/Abstract] OR "apoplexy"[Title/Abstract] OR "blood flow disturbance, brain"[Title/Abstract] OR "brain accident"[Title/Abstract] OR "brain attack"[Title/Abstract] OR "brain blood flow disturbance"[Title/Abstract] OR "brain insult"[Title/Abstract] OR "brain insultus"[Title/Abstract] OR "brain vascular accident"[Title/Abstract] OR "Brain Vascular Accidents"[Title/Abstract] OR "cerebral apoplexia"[Title/Abstract] OR "cerebral insult"[Title/Abstract] OR "cerebral stroke"[Title/Abstract] OR "Cerebral Strokes"[Title/Abstract] OR "cerebral vascular accident"[Title/Abstract] OR "cerebral vascular insufficiency"[Title/Abstract] OR "Cerebral Stroke"[Title/Abstract] OR "cerebro vascular accident"[Title/Abstract] OR "cerebrovascular accident"[Title/Abstract] OR "Cerebrovascular Accident, Acute"[Title/Abstract] OR "Cerebrovascular Accidents"[Title/Abstract] OR "Cerebrovascular Accidents, Acute"[Title/Abstract] OR "Cerebrovascular Apoplexy"[Title/Abstract] OR "cerebrovascular arrest"[Title/Abstract] OR "cerebrovascular failure"[Title/Abstract] OR "cerebrovascular injury"[Title/Abstract] OR "cerebrovascular insufficiency"[Title/Abstract] OR "cerebrovascular insult"[Title/Abstract] OR "Cerebrovascular Stroke"[Title/Abstract] OR "Cerebrovascular Strokes"[Title/Abstract] OR "Cerebrovascular Stroke"[Title/Abstract] OR "cerebrum vascular accident"[Title/Abstract] OR "cryptogenic stroke"[Title/Abstract] OR "CVA"[Title/Abstract] OR "insultus cerebralis"[Title/Abstract] OR "ischaemic seizure"[Title/Abstract] OR "ischemic seizure"[Title/Abstract] OR "stroke"[Title/Abstract] OR "Strokes"[Title/Abstract] OR "thrombotic stroke"[Title/Abstract] | 349162 |
| 4 | Stroke[MeSH Terms] | 180634 |
| 5 | "transcranial magnetic stimulation"[Title/Abstract] OR "Transcranial Magnetic Stimulations"[Title/Abstract] | 19976 |
| 6 | Transcranial Magnetic Stimulation[MeSH Terms] | 15579 |
| 7 | (#1 OR #2) AND (#3 OR #4) AND (#5 OR #6) | 3 |

## Table S2 Literature Search History of Embase

| Embase | | |
| --- | --- | --- |
| # | Query | Results |
| 1 | 'pain, shoulder':ti,ab,kw OR 'painful shoulder syndrome':ti,ab,kw OR 'pains, shoulder':ti,ab,kw OR 'shoulder pain':ti,ab,kw OR 'shoulder pains':ti,ab,kw | 12871 |
| 2 | shoulder pain'/exp | 22307 |
| 3 | 'acute cerebrovascular accident':ti,ab,kw OR 'acute cerebrovascular accidents':ti,ab,kw OR 'acute cerebrovascular lesion':ti,ab,kw OR 'acute focal cerebral vasculopathy':ti,ab,kw OR 'acute strokes':ti,ab,kw OR 'acute stroke':ti,ab,kw OR 'apoplectic stroke':ti,ab,kw OR 'apoplexia':ti,ab,kw OR 'apoplexy':ti,ab,kw OR 'blood flow disturbance, brain':ti,ab,kw OR 'brain accident':ti,ab,kw OR 'brain attack':ti,ab,kw OR 'brain blood flow disturbance':ti,ab,kw OR 'brain insult':ti,ab,kw OR 'brain insultus':ti,ab,kw OR 'brain vascular accident':ti,ab,kw OR 'brain vascular accidents':ti,ab,kw OR 'cerebral apoplexia':ti,ab,kw OR 'cerebral insult':ti,ab,kw OR 'cerebral strokes':ti,ab,kw OR 'cerebral vascular accident':ti,ab,kw OR 'cerebral vascular insufficiency':ti,ab,kw OR 'cerebral stroke':ti,ab,kw OR 'cerebro vascular accident':ti,ab,kw OR 'cerebrovascular accident':ti,ab,kw OR 'cerebrovascular accident, acute':ti,ab,kw OR 'cerebrovascular accidents':ti,ab,kw OR 'cerebrovascular accidents, acute':ti,ab,kw OR 'cerebrovascular apoplexy':ti,ab,kw OR 'cerebrovascular arrest':ti,ab,kw OR 'cerebrovascular failure':ti,ab,kw OR 'cerebrovascular injury':ti,ab,kw OR 'cerebrovascular insufficiency':ti,ab,kw OR 'cerebrovascular insult':ti,ab,kw OR 'cerebrovascular strokes':ti,ab,kw OR 'cerebrovascular stroke':ti,ab,kw OR 'cerebrum vascular accident':ti,ab,kw OR 'cryptogenic stroke':ti,ab,kw OR 'cva':ti,ab,kw OR 'insultus cerebralis':ti,ab,kw OR 'ischaemic seizure':ti,ab,kw OR 'ischemic seizure':ti,ab,kw OR 'stroke':ti,ab,kw OR 'strokes':ti,ab,kw OR 'thrombotic stroke':ti,ab,kw | 559691 |
| 4 | 'cerebrovascular accident'/exp | 452042 |
| 5 | 'transcranial magnetic stimulation':ti,ab,kw OR 'transcranial magnetic stimulations':ti,ab,kw | 28341 |
| 6 | 'transcranial magnetic stimulation'/exp | 33441 |
| 7 | (#1 OR #2) AND (#3 OR #4) AND (#5 OR #6) | 17 |

## Table S3 Literature Search History of Cochrane Library

| Cochrane Library | | |
| --- | --- | --- |
| # | Query | Results |
| 1 | “pain, shoulder” OR “painful shoulder syndrome” OR “Pains, Shoulder” OR “shoulder pain” OR “Shoulder Pains | 4664 |
| 2 | MeSH descriptor: [Shoulder Pain] explode all trees | 1461 |
| 3 | “Acute Cerebrovascular Accident” OR “Acute Cerebrovascular Accidents” OR “acute cerebrovascular lesion” OR “acute focal cerebral vasculopathy” OR “acute stroke” OR “Acute Strokes” OR “Acute Stroke” OR “apoplectic stroke” OR “apoplexia” OR “apoplexy” OR “blood flow disturbance, brain” OR “brain accident” OR “brain attack” OR “brain blood flow disturbance” OR “brain insult” OR “brain insultus” OR “brain vascular accident” OR “Brain Vascular Accidents” OR “cerebral apoplexia” OR “cerebral insult” OR “cerebral stroke” OR “Cerebral Strokes” OR “cerebral vascular accident” OR “cerebral vascular insufficiency” OR “Cerebral Stroke” OR “cerebro vascular accident” OR “cerebrovascular accident” OR “Cerebrovascular Accident, Acute” OR “Cerebrovascular Accidents” OR “Cerebrovascular Accidents, Acute” OR “Cerebrovascular Apoplexy” OR “cerebrovascular arrest” OR “cerebrovascular failure” OR “cerebrovascular injury” OR “cerebrovascular insufficiency” OR “cerebrovascular insult” OR “Cerebrovascular Stroke” OR “Cerebrovascular Strokes” OR “Cerebrovascular Stroke” OR “cerebrum vascular accident” OR “cryptogenic stroke” OR “CVA” OR “insultus cerebralis” OR “ischaemic seizure” OR “ischemic seizure” OR “stroke” OR “Strokes” OR “thrombotic stroke” | 91412 |
| 4 | MeSH descriptor: [Stroke] explode all trees | 17328 |
| 5 | “transcranial magnetic stimulation” OR “Transcranial Magnetic Stimulations” | 7659 |
| 6 | MeSH descriptor: [Transcranial Magnetic Stimulation] explode all trees 2589 | 2589 |
| 7 | (#1 OR #2) AND (#3 OR #4) AND (#5 OR #6) | 12 |

## Table S4 China National Knowledge Infrastructure

| China National Knowledge Infrastructure | | |
| --- | --- | --- |
| # | Query | Results |
| 1 | (肩痛 OR 肩手综合征) | 6148 |
| 2 | (卒中 OR 中风 OR 脑梗) | 204346 |
| 3 | (重复经颅磁刺激 OR rTMS OR 经颅磁刺激) | 6042 |
| 4 | (肩痛 OR 肩手综合征) AND (卒中 OR 中风 OR脑梗) | 3082 |
| 5 | (肩痛 OR肩手综合征) AND (重复经颅磁刺激 OR rTMS OR 经颅磁刺激) | 23 |
| 6 | (卒中 OR 中风 OR脑梗) AND (重复经颅磁刺激 OR rTMS OR 经颅磁刺激) | 1480 |
| 7 | (肩痛 OR肩手综合征) AND (卒中 OR 中风 OR脑梗) AND (重复经颅磁刺激 OR rTMS OR 经颅磁刺激) | 20 |

## Table S5 The eligibility criteria of subjects in included trials.

| **Study** | **Inclusion criteria** | **Exclusion criteria** |
| --- | --- | --- |
| Aydın 2024 | (1) adults (age between 18 and 70 years); (2) first-time stroke patients (ischemic or hemorrhagic/ cortical, subcortical or both) (diagnosed by cranial magnetic resonance imaging scan); (3) Presence of stroke in subacute or chronic stage; (4) Presence of subacute (duration: >one month) or chronic (duration: >three months) shoulder pain (length of time that patients had suffered from shoulder pain after stroke) (5) shoulder pain >4 on the Numeric Rating Scale (NRS) (6) Persistence of pain despite analgesic treatment for at least one week if analgesic treatment is being taken. | (1) if they had a clinical condition to be contraindicated for TMS; (2) history of any surgical intervention on the shoulder joint; (3) history of peri/intra-articular injection into the shoulder joint; (4) presence of any shoulder pathology such as rotator cuff injury or tendonitis, frozen shoulder, etc. diagnosed before stroke; (5) presence of a full-thickness rotator cuff tear visualized by musculoskeletal diagnostic ultrasound (US); (6) presence of upper limb muscles (teres major, subscapularis, pectoralis complex, subscapularis, brachioradialis, biceps, brachialis, pronator teres, flexor carpi radialis/ulnaris, flexor digitorum superficialis/profundus, flexor pollicis longus/brevis, lumbricales/interossei) > grade 3 spasticity defined according to the modified Ashworth Scale; (7) presence of severe cognitive impairment and aphasia; (8) history of malignancy or systemic rheumatic disease; (9) alcohol or drug addiction; (10) history of psychiatric illness such as major depression/personality disorder; (11) history of epilepsy or taking medication for epilepsy; (12) being diagnosed with dementia; (13) patients who have received TMS treatment before. In addition, patients who developed epilepsy during the study and did not regularly attend the study follow-up and treatment sessions, and patients with a new systemic problem or contraindication that would prevent the application of the treatment were excluded from the study. |
| Choi 2017 | (1) a history of stroke, (2) ≥ 6 months after stroke onset, (3) aged between 21–70 years, (4) hemiplegia after stroke, (5) significant shoulder pain with a minimal score of 4 on the Numeric Rating Scale (NRS, with 0 indicating no pain and 10 indicating the most severe pain) lasting for at least 3 months, (6) no change in the pain score on the NRS over 4 weeks despite pain medication (meloxicam and/or acetaminophen/tramadol hydrochloride), (7) limited passive range of motion (ROM) of a capsular pattern on physical examination, (8) no history of shoulder corticosteroid injections, (9) the absence of severe cognitive dysfunction or aphasia, and (10) the absence of contraindications for TMS such as a history of epileptic seizure, the presence of metal in the skull, or pacemaker placement. | We excluded patients who have central post-stroke pain or complex regional pain syndrome type I. All patients gave written informed consent. This study was conducted in compliance with The Code of Ethics of the World Medical Association (Declaration of Helsinki) for experiments involving humans. |
| Liu 2021 | （1）诊断符合《中国脑血管疾病诊治指南与共识（2016版）中的“脑 卒中诊断标准”且病灶部位经头颅 CT 或MRI证实；（2）符合《中国脑血管疾病防治指南》和《中国脑卒中康复治疗指南》规定肩手综合征标准的患者，年龄40~80岁，意识清楚，配合治疗，患者及家属知情并签署知情同意书；（3）均为首次发病，且病程≤3个月；（4）患肢休息时疼痛视觉模拟评分≥1 分，且患肢肿胀度较健侧未超过 50%；（5）未接受过相关的rTMS治疗。  Translation:  (1) The diagnosis was in accordance with the “Diagnostic Criteria for Cerebrovascular Disease” in the “China Cerebrovascular Disease Diagnosis and Treatment Guidelines and Consensus (2016 Edition)”, and the site of the lesion was confirmed by cranial CT or MRI; (2) patients with shoulder-hand syndrome were diagnosed in accordance with the “China Guidelines for the Prevention and Treatment of Cerebrovascular Disease” and “China Guidelines for Rehabilitation and Treatment of Cerebrovascular Disease”. The patients were 40-80 years old, conscious, cooperated with the treatment, and the patients and their families were informed and signed an informed consent form; (3) all of them had the first onset of the disease and the duration of the disease was ≤3 months; (4) the visual analog score of the pain in the affected limb at rest was ≥1, and the degree of swelling of the affected limb was not more than 50% compared with that of the healthy side; and (5) they did not receive the relevant rTMS treatment. | （1）体内植入金属者，如心脏 装有起搏器等；（2）有严重意识障碍或无法配合后续 治疗评估者；（3）既往有癫痫病史，或此次发病后有 癫痫发作者；（4）存在严重心、肝、肾等脏器功能损害 者；（5）病情呈进展性且颅内压明显增高者。  Translation:  (1) People with metal implants in their bodies, such as pacemakers in the heart; (2) People with severe impaired consciousness or who are unable to cooperate with subsequent therapeutic evaluations; (3) People with a history of epilepsy or who have epileptic seizures after the current episode; (4) People with severe cardiac, hepatic, renal, and other organ dysfunctions; and (5) People with progressive medical conditions and a significant increase in intracranial pressure. |
| LiuQ 2021 | （1）所有研究对象均符合1995年全国脑血管病会议诊断标准。（2）经影像学检查证实为脑卒中。（3）无意识障碍、无听力和言语障碍。（4）所有病人入院前未进行正规的康 复治疗。（5）有典型的SHS症状：①持续性疼痛，与任何刺激事件不成比例；②感觉：疼痛异常、疼痛过 敏；③血管收缩：皮温不对称、皮肤颜色双侧肢体不对称或改变（起初热、红，其后凉、发绀）；④水肿、出汗：水肿、出汗变化或不对称；⑤运动：肌力下降、肌张力障碍、关节活动度下降、运动功能障碍、震颤、 肌萎缩；⑥营养：皮肤（变薄、发亮、溃烂）、指甲（过长、脆甲症）、汗毛增多（减少）、骨质疏松。  Translation:  (1) All study subjects met the diagnostic criteria of the 1995 National Conference on Cerebrovascular Disease. (2) Stroke was confirmed by imaging examination. (3) There was no consciousness disorder, hearing or speech disorder. (4) None of the patients had received formal rehabilitation treatment before admission. (5) Typical symptoms of SHS: ①persistent pain, disproportionate to any stimulus event; ②sensation: pain abnormality, pain hypersensitivity; ③vasoconstriction: asymmetrical skin temperature, asymmetry or change in skin color of both limbs (hot and red at first, then cool and cyanotic); ④edema and sweating: edema and sweating change or asymmetry; ⑤movement: loss of muscle strength, dystonia, and decrease in joint mobility, motor dysfunction, tremor, muscular atrophy; ⑥ Nutrition: skin (thinning, shiny, ulcerated), nails (excessive length, brittle nail syndrome), increased (decreased) sweat hair, osteoporosis. | （1）卒中前新发肩周炎；（2）有风湿、类风湿关节炎病史；（3）痛风性关节炎反复发作病史；（4）病程中病侧上肢有外伤史。Translation:  (1) new onset of frozen shoulder before stroke; (2) history of rheumatoid or rheumatoid arthritis; (3) history of recurrent gouty arthritis; and (4) history of trauma to the diseased side of the upper extremity during the course of the disease. |
| Tao 2023 | ①符合《中国各类主要脑血管病诊断要点2019》，并通过头颅CT或MRI 证实；②首次发病，年龄≤80 岁者；③病程≤6个月，生命体征稳定，无认知障碍，可以配合治疗；④脑卒中后偏瘫；⑤活动或静息状态下 存在偏瘫侧肩痛，数字疼痛评分法（numerical pain rating scale，NPRS）≥4分；⑥无rTMS禁忌，如癫痫发作史、颅骨内金属物或放置起搏器等。  Translation:  ① Patients comply with the “Diagnostic Points of Various Major Cerebrovascular Diseases in China 2019”, and confirmed by cranial CT or MRI; ② first onset of disease, age ≤ 80 years old; ③ duration of the disease ≤ 6 months; vital signs are stable; there is no cognitive impairment, and can be cooperated with the treatment; ④ post-stroke hemiparesis; ⑤ Presence of shoulder pain on the hemiplegic side with a numerical pain rating scale (NPRS) ≥4 in active or resting state; ⑥ No contraindications to rTMS, such as history of seizures, intracranial metal objects, or placement of pacemakers. | ①病情不稳定或继发性脑卒中；②发病前曾有肩关节相关的肌肉骨骼系统疾病；③年龄>80岁；④病程>6个月；⑤存在意识障碍，言语障碍、认知障碍以及精神障碍等影响评估和治疗；⑥有rTMS禁忌。  Translation:  (1) unstable condition or secondary stroke; (2) previous shoulder-related musculoskeletal disorders prior to the onset of the disease; (3) age >80 years; (4) duration of the disease >6 months; (5) the presence of disorders of consciousness, speech disorders, cognitive disorders, and psychiatric disorders that interfere with the assessment and treatment; and (6) contraindications to rTMS. |
| Tian 2021 | （1）符合 2019年中华医学会 神经病学分脑血管病学组发布的《中国各类主要脑血管病诊断要点》中关于脑卒中诊断标准；（2）所有患者均符合 SHS“布达佩斯诊断标准”，即同时符合ⅰ、ⅱ、ⅲ项，ⅰ即与伤害事件不成比例的剧痛、持续性疼痛，ⅱ必须包括感觉［有感觉过敏和（或）超敏］、血管收缩［有皮肤温度增高和（或）皮肤颜色红肿］、患侧上肢出汗异常（或）水肿［有水肿和（或） 出汗异常］、运动/营养［关节活动范围受限和（或）肌肉运动功能障碍和（或）皮肤、毛发营养性不良］4项中任意1项，ⅲ即其他诊断不能更好地解释此症状和体征。  Translation:  (1) Patients meet the diagnostic criteria for stroke in the “Diagnostic Points for Various Major Cerebrovascular Diseases in China” issued by the Cerebrovascular Disease Group of the Neurology Division of the Chinese Medical Association in 2019; (2) All patients meet the SHS “Budapest Diagnostic Criteria”, which means that they meet the items ⅰ, ⅱ, and ⅲ at the same time, ⅰ, i.e., severe pain that is disproportionately associated with the injury event, persistent pain, ⅱ must include any one of the four items: sensation [sensory hypersensitivity and/or hypersensitivity], vasoconstriction [increased skin temperature and/or redness of the skin color], abnormal sweating (or edema) of the affected side of the upper extremity [edema and/or abnormal sweating], motor/nutritional [limited range of motion of joints and/or muscular movement dysfunction and/or dystrophy of the skin and hair], and ⅲ that the symptoms are not better explained by other diagnoses. Other diagnoses do not better explain this symptom and sign. | （1）严重心脏病及装有心脏起搏器患者；（2）颈部及以上部位有非钛合金金属异物植入患者；（3）有脑部手术或癫痫病史患者；（4）肿瘤患者；（5）认知障碍患者；（6）服用止痛药物或接受其他物理方法治疗疼痛患者；（7）患侧上肢肌张力高，不能伸直放入水槽测前臂肿胀者。  Translation:  (1) patients with severe heart disease and pacemakers; (2) patients with non-titanium metal foreign body implants in the neck and above; (3) patients with brain surgery or a history of epilepsy; (4) patients with tumors; (5) patients with cognitive disorders; (6) patients who are taking pain medications or undergoing other physical methods of treating their pain; and (7) patients who have high muscle tone in the upper extremity of the affected side and are unable to straighten it into the sink to measure forearm swelling. |
| Wang 2017 | 同时满足1)《中国脑血管病防治指 南》(2010版)诊断标准确诊为脑卒中(脑梗死或脑出血)；2)单侧肢体发病、主诉患侧肩痛；3)发病后无神志异常，治疗合作；4)年龄20~80岁；5)患者与家属同意接受入组试验；6)签署知情同意书。  Translation:  The patients must also meet the following criteria: 1) diagnosis of stroke (cerebral infarction or cerebral hemorrhage) according to the 2010 edition of the Chinese Guidelines for the Prevention and Treatment of Cerebrovascular Disease; 2) unilateral onset of the disease, with the complaint of shoulder pain on the affected side; 3) no mental abnormality after the onset of the disease, and cooperation with the treatment; 4) age of 20-80 years old; 5) consent of the patients and their family members to be enrolled in the trial; and 6) signing of the informed consent form. | 1 )有癫痫、房颤病史；2)心力衰竭及危重患者；3)肱骨骨折、锁骨骨折；4)佩戴心脏起搏器者；5)妊娠期妇女；6)有出血倾向患者；7 )既往有肩关节疼痛病史或相关疾病，如肩周炎。  Translation:  1 ) history of epilepsy and atrial fibrillation; 2) heart failure and critically ill patients; 3) humerus fracture and clavicle fracture; 4) pacemaker wearers; 5) women during pregnancy; 6) patients with bleeding tendency; and 7 ) previous history of shoulder pain or related diseases, such as frozen shoulder. |
| Xiang 2015 | ①根据《中国脑血管病防治指南》（2010版）诊断标准确诊为脑卒中（脑梗死或脑出血）；②患侧上肢存在功能障碍；③主诉有患侧肩关节疼痛；④因肩痛在我院进行过局部超声检查。  Translation:  ① Diagnosis of stroke (cerebral infarction or cerebral hemorrhage) was confirmed according to the diagnostic criteria of the Chinese Guidelines for the Prevention and Treatment of Cerebrovascular Disease (2010 edition); ② functional impairment of the upper extremity of the affected side existed; ③ complaint of pain in the shoulder joint of the affected side existed; ④ local ultrasonography had been performed in our hospital because of shoulder pain. | ①既往已存在肩痛症状或已确诊为相关疾病，如肩袖损伤、肩关节骨折脱位等；②既往有患侧肩关节手术史；③存在意识障碍或严重精神疾病等肩痛无法确认的情况。  Translation:  ① Pre-existing symptoms of shoulder pain or confirmed diagnosis of related diseases, such as rotator cuff injury, shoulder fracture dislocation, etc.; ② Pre-existing history of shoulder surgery on the affected side; ③ Presence of disorders such as disorders of consciousness or serious mental illnesses that cannot be confirmed by shoulder pain. |
| Xue 2019 | 无 | 无 |
| Zhang 2019 | ①符合《中国急性缺血性脑卒中诊治指南2014脑梗死》和《各类脑血管疾病诊断要点》脑出血的诊断标准；②签署同意书。  Translation:  ① Patients meet the diagnostic criteria for cerebral hemorrhage in the Chinese Guidelines for Diagnosis and Treatment of Acute Ischemic Stroke 2014 Cerebral Infarction and Diagnostic Points for Various Cerebrovascular Diseases; ② Patients sign the consent form. | ①房颤、癫痫患者；②严重心脏功能障碍患者，比如心力衰竭；③装有心脏起搏器或者体内有钢板患者；④出血倾向患者；⑤有肩周炎或者肩关节疼痛病史患者。  Translation:  Patients with atrial fibrillation or epilepsy; patients with severe cardiac dysfunction, such as heart failure; patients with pacemakers or plates; patients with bleeding tendency; and patients with a history of frozen shoulder or shoulder joint pain. |
| Zhen 2022 | 符合CS诊断标准；患侧上肢出现肢体功能障碍；首次发病；意识清醒，认知功能正常；知情且自愿签署同意书。  Translation:  Patients meets diagnostic criteria for CS; Patients develop limb dysfunction in the affected upper limb; First onset; Consciousness and normal cognitive function; Patients voluntarily signed consent form. | 病情恶化，再次出现脑出血、脑梗死；合并精神障碍、视听障碍；合并心、肝、肾等功能障碍；合并恶性肿瘤疾病；体内存在金属植入物；存在癫痫史；既往存在肩痛症状，或合并肩部疾病；存在患侧肩关节手术史。  Translation:  Deterioration of the disease, re-occurrence of cerebral hemorrhage, cerebral infarction; comorbid mental disorders, visual and auditory disorders; comorbid cardiac, hepatic and renal dysfunction; comorbid malignant neoplastic diseases; presence of metallic implants in the body; a history of epilepsy; presence of shoulder pain symptoms in the past, or comorbid with shoulder disorders; a history of surgery of the affected shoulder joint. |
